# Monomeric C-reactive protein is associated with severity and prognosis of decompensated hepatitis B cirrhosis

**DOI:** 10.3389/fimmu.2024.1407768

**Published:** 2024-06-04

**Authors:** Ning Gao, Ping Yuan, Zhao-Ming Tang, Jia-Geng Lei, Ze-Rui Yang, Mustafa Ahmed, Zhen-Yu Yao, Dan Liang, Yi Wu, Hai-Yun Li

**Affiliations:** ^1^ Department of Infectious Disease, The Second Affiliated Hospital of Xi’an Jiaotong University, Xi’an, China; ^2^ Ministry of Education (MOE) Key Laboratory of Cell Activities and Stress Adaptations, School of Life Sciences, Lanzhou University, Lanzhou, China; ^3^ Department of Biochemistry and Molecular Biology, School of Basic Medical Sciences, Xi’an Jiaotong University, Xi’an, China; ^4^ Ministry of Education (MOE) Key Laboratory of Environment and Genes Related to Diseases, School of Basic Medical Sciences, Xi’an Jiaotong University, Xi’an, China; ^5^ Department of Physiology, Gansu University of Chinese Medicine, Lanzhou, China

**Keywords:** C-reactive protein, monomeric C-reactive protein, autoantibodies, decompensated hepatitis B cirrhosis, inflammation

## Abstract

C-reactive protein (CRP) is an acute-phase protein produced by the liver in response to infection and during chronic inflammatory disorders. Systemic inflammation is a major driver of cirrhosis progression from the compensated to the decompensated stage. Previous studies have shown that pentameric CRP (pCRP) to be a weak predictor of disease severity and prognosis in patients with decompensated hepatitis B cirrhosis, with it being only helpful for identifying patients with a higher short-term risk of death under certain conditions. Accumulating evidence indicates that pCRP dissociates to and acts primarily as the monomeric conformation (mCRP) at inflammatory loci, suggesting that mCRP may be a potentially superior disease marker with higher specificity and relevance to pathogenesis. However, it is unknown whether mCRP and anti-mCRP autoantibodies are associated with disease severity, or progression in decompensated hepatitis B cirrhosis. In this study, we evaluated the serum levels of mCRP and anti-mCRP autoantibodies in patients with decompensated cirrhosis of hepatitis B and their association with disease severity and theoretical prognosis. The results showed that patients with high mCRP and anti-mCRP autoantibody levels had more severe liver damage and that coagulation function was worse in patients with high anti-mCRP autoantibodies. Analysis of the correlation between pCRP, mCRP and anti-mCRP autoantibody levels with Model for End-Stage Liver Disease (MELD), Albumin-Bilirubin (ALBI), and Child–Turcotte–Pugh (CTP) prognostic scores showed that mCRP was the most strongly correlated with MELD score, followed by anti-mCRP autoantibodies; conversely, pCRP was not significantly correlated with prognostic score. Therefore, mCRP and anti-mCRP autoantibodies may be more advantageous clinical indicators than pCRP for evaluating the pathological state of decompensated hepatitis B cirrhosis.

## 1 Introduction

Hepatitis B virus (HBV) infection is one of the principal causes of cirrhosis (1). As it progresses, hepatitis B cirrhosis can enter the more severe stage of decompensated hepatitis B cirrhosis (2), which involves serious complications, such as gastrointestinal hemorrhage, hepatic encephalopathy, and ascites (3). In the Asia-Pacific region, hepatitis B virus infection is also a major cause of acute-on-chronic liver failure (ACLF) (4). Such patients with ACLF encompass a dysregulated, systemic inflammatory response, which can precipitate extra hepatic organ failures. It is believed that inflammation has an important role in the pathogenesis and progression of hepatitis (5). Pathogen-associated molecular patterns and damage-associated molecular patterns can activate the intrinsic defense mechanisms of the liver and provoke an inflammatory response (6). Tissue damage caused by chronic inflammation may lead to increased circulating levels of damage-associated molecular patterns, thus aggravating the immune response and resulting in hepatocyte injury and necrosis (7, 8).

Pentameric C-reactive protein (pCRP) is a major acute-phase protein secreted by the liver (9). The plasma concentration of pCRP increases dramatically after tissue injury (10, 11), and it is often used as a nonspecific clinical marker of inflammation (12). pCRP recognizes the ligand phosphorylcholine on the surface of pathogens or damaged cells (13) and regulates activation of the complement system by recruiting C1q (14, 15), thus behaving similarly to pattern recognition receptors that may have key roles in innate immunity. Notably, there is increasing evidence indicating that pCRP can de-polymerize locally to produce monomeric CRP (mCRP) (15, 16), which was first characterized and conceptualized by Potempa et al. in 1983 (17). Structural intermediates with impaired pentameric assembly but a near-native subunit structure, such as mCRPm and pCRP* (18), have also been identified during the conversion of the pCRP to the fully activated conformation mCRP (19). Monomeric CRP can stimulate cellular inflammatory responses (20–22), modulate the activation of the complement system (23), and regulate low-density lipoprotein metabolism (24) with significantly greater efficiency. Thus, it is likely that mCRP represents a “functional” isoform with significantly increased activity (19). Studies of CRP and its autoantibodies have found that some functional anti-CRP autoantibodies do not interact with pCRP; instead, they specifically recognize mCRP (25), suggesting that CRP function is dependent on conformational change (26).

CRP is also a clinical indicator used to evaluate the condition and progression of hepatitis B (27–29). However, it remains unknown whether mCRP and anti-mCRP autoantibodies correlate with disease severity or are predictive of its progression. During the present study, 166 patients with decompensated hepatitis B cirrhosis were identified. The serum pCRP, mCRP, anti-mCRP autoantibodies, and anti-a.a.35-47 autoantibodies (26) were correlated with major liver-associated clinical indicators and compared using different prognostic scoring models and correlation indicators. The aim of the present study was to evaluate whether mCRP, anti-mCRP autoantibodies, and anti-a.a.35-47 autoantibodies are associated with disease severity, to evaluate their predictive value for the progression of liver disease, and to provide a potential basis for the discovery of more sensitive and effective clinical biomarkers.

## 2 Materials and methods

### 2.1 Patient selection

The population of this clinical study comprised 166 patients with HBV-decompensated cirrhosis hospitalized in the Department of Infectious Diseases of the Second Affiliated Hospital of Xi’an Jiaotong University between April 2021 and October 2021 and 96 healthy controls. The clinical data recorded included basic characteristics (age, sex), history of chronic illness, clinical presentation, complications, serological indicators at admission (complete blood count, liver biochemistry, coagulation indicators), immunological indicators, liver ultrasound results, and abdominal computed tomography results. The plasma samples were collected from these patients before therapy initiation. HBV-decompensated cirrhosis inclusion criteria: the cause of hepatitis B is clearly diagnosed, and the diagnosis of decompensated cirrhosis complies with the diagnostic criteria of the “Guidelines for the Diagnosis and Treatment of Liver Cirrhosis” formulated by the Infectious Diseases Branch and the Hepatology Branch of the Chinese Medical Association in 2019, and age 18-90 years old. HBV-decompensated cirrhosis exclusion criteria were: (1) Chronic liver disease of non-HBV etiology; (2) HBV coinfected with hepatitis A, C, D, E virus or combined with other etiologies, such as nonalcoholic or alcoholic steatohepatitis; (3) age< 18 years; (4) pregnancy; (5) thyroid disease; (6) previous chronic renal disease; (7) coronary heart disease treated with an anticoagulant; (8) hepatocellular carcinoma or other types of tumor; (9) liver transplantation; (10) severe immunosuppression due to active tuberculosis, human immunodeficiency virus infection, or hematological disease; and (12) reception of a transfusion before admission. This study was approved by the Ethics Committee of Xi’an Jiaotong University (2018059), and the study was conducted in accordance with the Declaration of Helsinki. All study participants fully understood the purpose of the study and provided informed consent in writing to voluntarily participate in the study. The Child–Turcotte–Pugh (CTP) score, Model for End-Stage Liver Disease (MELD) score, albumin-bilirubin (ALBI) score, CAR, CRP/pre-albumin (pre-Alb), and AST/ALT (AAR) were calculated based on the clinical presentation and biochemical test results of patients with decompensated cirrhosis at the time of admission.

#### 2.1.1 ELISA assay quantifying mCRP and anti-mCRP autoantibody

Detection of mCRP and anti-mCRP autoantibody was performed as described (26, 30). Briefly, anti-human CRP mAb CRP-8 (0.3 µl/ml) and mCRP (2 µg/ml) were immobilized onto microtiter wells in a coating buffer (10 mM sodium carbonate/bicarbonate, pH 9.6) overnight at 4˚C. After each incubation step, wells were washed three times with TBS containing 0.02% NP-40. Wells were then blocked with blocking buffer (1% BSA in TBS) for 1 h. Samples diluted in blocking buffer were added into wells for 1 h. Captured mCRP was detected with a sheep anti-human CRP polyclonal antibody (1:2,000 in blocking buffer), followed by detection with an HRP-labeled donkey anti-sheep IgG (1:20,000 in blocking buffer). Captured anti-mCRP autoantibody was detected with an HRP-labeled mouse anti-human IgG (1:20,000 in blocking buffer). Wells were incubated with TMB buffer for 30 min and stopped with 1 M H_2_SO_4_. Absorbance at OD570 and OD450 nm was measured with a microplate reader. The OD value of each sample was calculated as OD450-OD570 nm. A total of 100 µl volume was used at all incubation steps, while 300 µl volume was used for washing after each incubation step.

#### 2.1.2 CTP score

The CTP scoring system was used to calculate the total score after assigning scores to the following five parameters: serum albumin, total bilirubin, prothrombin time, ascites, and hepatic encephalopathy. The severity of ascites comprising the CTP score was determined by imaging, and the severity of hepatic encephalopathy was determined from medical records (31). These scores were summed to obtain the CTP score (**Table 1**).

**Table 1:** CTP scores.

Clinical or biochemical indicator	Score		
	1	2	3
**Hepatic encephalopathy**	**No**	**I~II**	**III~IV**
**Ascites**	**No**	**Mild**	**Moderate/severe**
**TBIL (μmol/L)**	**<34**	**34~50**	**>50**
**Alb (g/L)**	**>35**	**28~35**	**<28**
**Prolonged PT (s)**	**<4**	**4~6**	**>6**

Alb, albumin; CTP, Child–Turcotte–Pugh; PT, prothrombin time; TBIL, total bilirubin.

#### 2.1.3 MELD score

The MELD score for evaluating decompensated hepatitis B cirrhosis was calculated using the following formula:


MELD score = 3.78 × ln [TBIL (μmol/L) ÷ 17.1] + 11.2 × ln [INR] + 9.57 × ln [Cr (μmol/L) ÷ 88.4] + 6.43

Where TBIL represents total bilirubin, INR represents the international normalized ratio, and Cr represents creatinine (32).

#### 2.1.4 ALBI score

The ALBI score was initially used to assess survival after surgical treatment for patients with chronic liver disease and concomitant hepatocellular carcinoma. However, it has been found to be predictive of the 3-month survival of patients with HBV-associated acute-on-chronic liver failure.

The ALBI score was calculated using the following formula:


ALBI = − 0.085 × [Alb (g/L)] + 0.66 × Lg [TBIL (μmol/L)]

where Alb represents albumin, and TBIL represents total bilirubin.

ALBI scores are classified into the following three grades: grade 1, ALBI<-2.60, indicating a good prognosis; grade 2, ALBI ≥-2.60 but ≤-1.39, indicating a moderate prognosis; and grade 3, ALBI >-1.39, indicating a poor prognosis (33).

#### 2.1.5 AAR= AST/ALT

### 2.2 Statistical analyses

R (4.1.3) and GraphPad Prism 9 were used for statistical analyses. Measures with normal distribution were expressed as the mean ± standard deviation, and measures with non-normal distribution were expressed as the median and interquartile range. The t-test was performed to compare groups of data with normal distribution. The Mann–Whitney U test was performed to compare groups of data with non-normal distribution. Count data were described as percentages. Correlations were assessed using Spearman’s correlation analysis (correlation coefficient r). The prognostic value was assessed using the Kaplan–Meier analysis and the log-rank test. Differences with *p*< 0.05 were considered statistically significant.

## 3 Results

### 3.1 General data of patients with hepatitis B decompensated cirrhosis

Of the 166 patients with decompensated cirrhosis, 116 (69.9%) were male, for a male-to-female ratio of 2.4:1. The mean age of the patients was 53 ± 10 years. The average length of hospitalization was 9-15 days. **Table 2** details the characteristics of the 166 patients with decompensated hepatitis B cirrhosis.

**Table 2:** General data of patients with hepatitis B decompensated cirrhosis.

Clinical evaluation	Number of patients N=166
Male sex, N (%)	116 (69.9)
Age (year)	53 ± 10
Length of hospital stay, days median (range)	12 (9,15)
>14	67 (40.4)
<=14	99 (59.6)
Analytical variables	Median (range)
TBIL (μmol/L)	20.95 (14.4, 35.55)
ALT (IU/L)	27 (19,42)
AST (IU/L)	37 (26.75,53)
Alb (g/L)	35.95 (29.9,39.7)
pre-Alb (mg/L)	140 (90,197)
Serum Creatinine (μmol/L)	52.445 (43.272,62.778)
Na (mmol/L)	139.4 (137.85,140.675)
PT (s)	12.5 (11.15,14.1)
PTR	1.12 (1,1.27)
INR	1.13 (1.005,1.31)
PT%	78.2 (63.2,95.45)
APTT (s)	31. 3(25.65,38.5)
FIB (g/L)	1.96 (1.49,2.46)
white blood cell count (10^9/L)	3.765 (2.3925,4.9725)
platelet count (10^9/L)	91.5 (59,150.5)
neutrophil count (10^9/L)	2.02 (1.3825,2.97)
monocyte count (10^9/L)	0.295 (0.1825,0.4)
lymphocyte count (10^9/L)	1.01 (0.6,1.5375)

TBIL, total bilirubin; ALT, alanine aminotransferase; AST, aspartate aminotransferase; Alb, serum albumin; PT, prothrombin time; PTR, prothrombin ratio; INR, international normalized ratio; PT%, prothrombin ratio; APTT, activated partial thromboplastin time; FIB, fibrinogen. The quantitative data were expressed as median M (P25, P75).

### 3.2 Plasma levels of pCRP/mCRP/anti-mCRP autoantibodies and anti-a.a.35-47 autoantibodies

**Figure 1:**
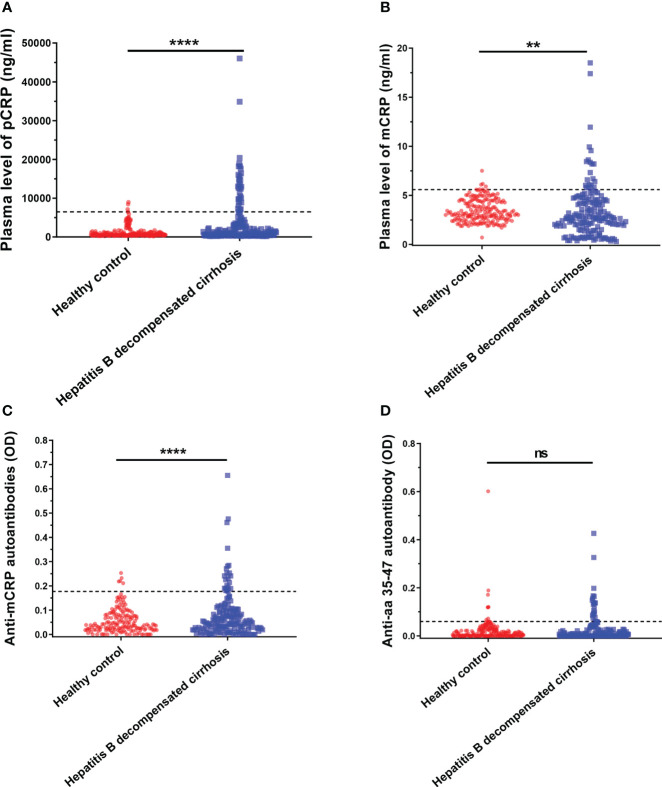
Serum pCRP **(A)**, mCRP **(B)**, anti-mCRP autoantibody **(C)**, and anti-a.a.35-47 autoantibody **(D)** levels of patients with decompensated hepatitis B cirrhosis. The dashed lines in the figure represent cut-off values determined at 95% for the healthy population using the one-sided percentile method. Serum CRP, mCRP, and anti-mCRP autoantibody levels were significantly higher in the decompensated hepatitis B cirrhosis patient group than in the healthy control group. The difference in a.a.35-47 autoantibodies was not statistically significant. **p<0.01; ****p<0.0001; ns, not significant.

First, serum samples were collected from age- and sex-matched healthy individuals and patients with decompensated cirrhosis. Serum pCRP, mCRP, anti-mCRP autoantibody, and anti-a.a.35-47 autoantibody levels in each group were measured, and the differences in the serum concentrations of the four indicators between patients with decompensated hepatitis B cirrhosis and healthy individuals were compared (**Figure 1**).

It has been previously reported that a.a.35-47 is expressed only after pCRP is dissociated into the mCRP conformation (34, 35). Since antibody production depends on the presentation and recognition of intact antigen, the detection of autoantibodies specifically recognizing mCRP under pathological conditions in decompensated cirrhosis would provide strong support for conditional activation *in vivo*. Thus, in addition to serum pCRP and mCRP levels, anti-mCRP autoantibodies and anti-a.a.35-47 autoantibodies were also used as indicators. The results are shown in **Figure 1**, where the dashed line represents the cut-off value, determined at 95% using one-sided percentiles in the normal population. The results showed that serum CRP (*p*< 0.0001), mCRP (*p* = 0.0012), and anti-mCRP autoantibody (*p<* 0.0001) levels in some decompensated hepatitis B cirrhosis patients were significantly higher than in the normal group; there was no significant difference in anti-a.a.35-47 autoantibody levels. Since the incidence of follow-up endpoint events in patients with decompensated hepatitis B cirrhosis was less than 10%, direct prognostic analysis could not be performed. Therefore, we used serum cut-off values to further subdivide patients into high- and low-level groups. The associations and differences between serum and clinical indicators and prognostic scores of patients in different groups were observed. In addition, because anti-a.a.35-47 autoantibody levels in the decompensated hepatitis B cirrhosis group did not differ significantly from that of the normal group (**Figure 1D**), it was not included in subsequent analyses.

### 3.3 Comparative analysis of clinical and laboratory data between patients with high mCRP/anti-mCRP autoantibody levels with low mCRP/anti-mCRP autoantibody levels

First, liver function-related indicators, including total bilirubin (TBIL), alanine transaminase (ALT), aspartate transaminase (AST), albumin (Alb), pre-albumin (pre-Alb), creatinine (Cre), and sodium (Na), were selected as clinical correlation indicators. Second, patients with chronic liver disease and cirrhosis often have many abnormalities in hemostatic and coagulation indicators, such as decreased platelet count, prolonged prothrombin time (PT), and decreased fibrinogen levels. Therefore, the coagulation-related indicators PT (s), PTR, INR, PTA, APTT (s), and FIB (g/L) can be selected as evidence for supporting the status and prognosis of liver disease. Finally, since CRP is typically associated with inflammation, white blood cell count, neutrophil count, monocyte count, and lymphocyte count were inflammatory cell-related indicators included as references for evaluating the status of inflammation in the body.

The results showed that the high mCRP level group had higher AST, TBIL, and Cre and lower Alb, pre-Alb, and serum sodium at admission than the low mCRP group. The high anti-mCRP autoantibody group had higher ALT and AST compared to the low anti-mCRP autoantibody group. Overall, patients in the high mCRP and autoantibody groups had reduced liver function to varying degrees. The high anti-mCRP autoantibody group exhibited differences in coagulation indicators compared to the low anti-mCRP autoantibody group, primarily in higher PTR and INR and lower PTA and FIB, as well as several indicators that fell outside the normal reference range, indicating decreased coagulation function in patients with high anti-mCRP autoantibody levels. There was no correlation between serum mCRP levels and coagulation-related indicators. There was no correlation between serum mCRP or anti-mCRP autoantibody levels and complete blood count indicators (**Tables 3**, **4**).

**Table 3:** Comparison of clinical data between patients with high mCRP and low mCRP levels.

	mCRP		*p- value*
	high	low	
TBIL (μmol/L)	44.7 (18.73,64.73)	20.2 (13.7,29.8)	0.0155*
ALT (IU/L)	35.5 (23.75,60.5)	26 (18,38.5)	0.0693
AST (IU/L)	46.5 (33.25,84.75)	37 (26,51)	0.0183*
Alb (g/L)	30.65 (28.1,36.18)	36.1 (30.1,40.15)	0.0194*
pre-Alb (mg/L)	85 (56,151)	142 (98.5,199)	0.0248*
Cre (μmol/L)	61.03 (55.74,69.79)	51.07 (43.01,59.4)	0.0039**
Na (mmol/L)	137.8 (135,139.3)	139.5 (138.7,140.7)	0.0094**
PT (s)	13.7 (12.1,15)	12.3 (11,13.8)	0.0624
PTR	1.19 (1.02,1.35)	1.11 (0.99,1.25)	0.3001
INR	1.25 (1.09,1.37)	1.11 (0.99,1.27)	0.1097
PT%	65.1 (56.4,82.9)	80.2 (64.75,97)	0.1019
APTT (s)	34.9 (29.1,42.7)	31.2 (25.6,38.35)	0.0740
FIB (g/L)	1.59 (1.39,2.44)	1.94 (1.52,2.46)	0.7201
pCRP (ng/ml)	4777.3(1408.74,15022.73)	919.13(446.66,2916.03)	0.0014*
anti-mCRP (OD)	0.09(0.06,0.19)	0.05(0.03,0.1)	0.0066*
anti-a.a.35-47 (OD)	0.02(0,0.08)	0.01(0,0.02)	0.0683
white blood cell count (10^9/L)	4.07 (3.4,4.48)	3.73 (2.35,4.98)	0.3155
platelet count (10^9/L)	115 (84,140)	90 (59,154)	0.4886
neutrophil count (10^9/L)	2.24 (1.75,2.83)	1.94 (1.35,2.97)	0.20
monocyte count (10^9/L)	0.33 (0.24,0.45)	0.28 (0.17,0.38)	0.0747
lymphocyte count (10^9/L)	1.24 (0.95,1.4)	0.99 (0.56,1.55)	0.3537

TBIL, total bilirubin; ALT, alanine aminotransferase; AST, aspartate aminotransferase; Alb, serum albumin; PT, prothrombin time; PTR, prothrombin ratio; INR, international normalized ratio; PT%, prothrombin ratio; APTT, activated partial thromboplastin time; FIB, fibrinogen. The quantitative data were expressed as median M (P25, P75). The cut-off value is calculated using the 95% unilateral percentile method of the normal population. Serum test values ≥ cut-off values are in the high mCRP group, and serum test values< cut-off values are in the low mCRP group. *p<0.05; **p<0.01.

**Table 4:** Comparison of clinical data between patients with high anti-mCRP autoantibodies and low anti-mCRP autoantibodies.

	anti-mCRP autoantibodies		*p- value*
	high	low	
TBIL (μmol/L)	25.9(19.1,38.36)	20.4(13.86,34.8)	0.2714
ALT (IU/L)	28(20.75,60)	26.5(18,39.5)	0.0057**
AST (IU/L)	52.5(36,107)	34.5(26,50.25)	0.0174*
Alb (g/L)	30(27.93,35.23)	36.65(31.28,40.35)	0.7133
pre-Alb (mg/L)	97.5(53.5,115.75)	150(99,205)	0.8641
Cre (μmol/L)	53.24(45.39,60.67)	52.36(43.25,62.65)	0.6909
Na (mmol/L)	137.4(135.05,138.35)	139.6(138.7,140.8)	0.5965
PT (s)	13.9(12.98,14.93)	12.3(11,13.8)	0.0536
PTR	1.25(1.1,1.32)	1.11(0.99,1.25)	0.0168*
INR	1.28(1.16,1.36)	1.11(0.99,1.27)	0.0032**
PT%	64.75(56.85,78.7)	80.2(64.75,97.9)	0.0161*
APTT (s)	35.55(33.2,40.73)	29.8(25.55,38.2)	0.0707
FIB (g/L)	1.53(1.41,2.05)	2.03(1.53,2.48)	0.0144*
pCRP(ng/ml)	2885.34 (1022.35,7157.73)	990.85 (497.36,3453.86)	0.0433*
mCRP(ng/ml)	3.61 (2.77,5.47)	2.64 (1.63,4.31)	0.0001*
anti-a.a.35-47 (OD)	0.13 (0.09,0.16)	0.001 (0,0.014)	0.0611
white blood cell count (10^9/L)	3.31(2.37,4.55)	3.88 (2.39,5.06)	0.3670
platelet count (10^9/L)	80(51.5,126)	93 (59.75,150.5)	0.2820
neutrophil count (10^9/L)	1.81(1.09,2.37)	2.04 (1.42,2.98)	0.4320
monocyte count (10^9/L)	0.32(0.19,0.45)	0.28 (0.18,0.4)	0.5887
lymphocyte count (10^9/L)	0.92(0.53,1.35)	1.02 (0.62,1.55)	0.4012

TBIL, total bilirubin; ALT, alanine aminotransferase; AST, aspartate aminotransferase; Alb, serum albumin; PT, prothrombin time; PTR, prothrombin ratio; INR, international normalized ratio; PT%, prothrombin ratio; APTT, activated partial thromboplastin time; FIB, fibrinogen. The quantitative data were expressed as median M (P25, P75). The cut-off value is calculated using the 95% unilateral percentile method of the normal population. Serum test values ≥ cut-off values are in the high anti-mCRP autoantibodies group, and serum test values< cut-off values are in the low anti-mCRP autoantibodies group. *p<0.05; **p<0.01.

### 3.4 Association between CRP, mCRP, anti-mCRP autoantibodies, and anti-a.a.35-47 autoantibodies and MELD, CTP, and ALBI prognostic scores

**Figure 2:**
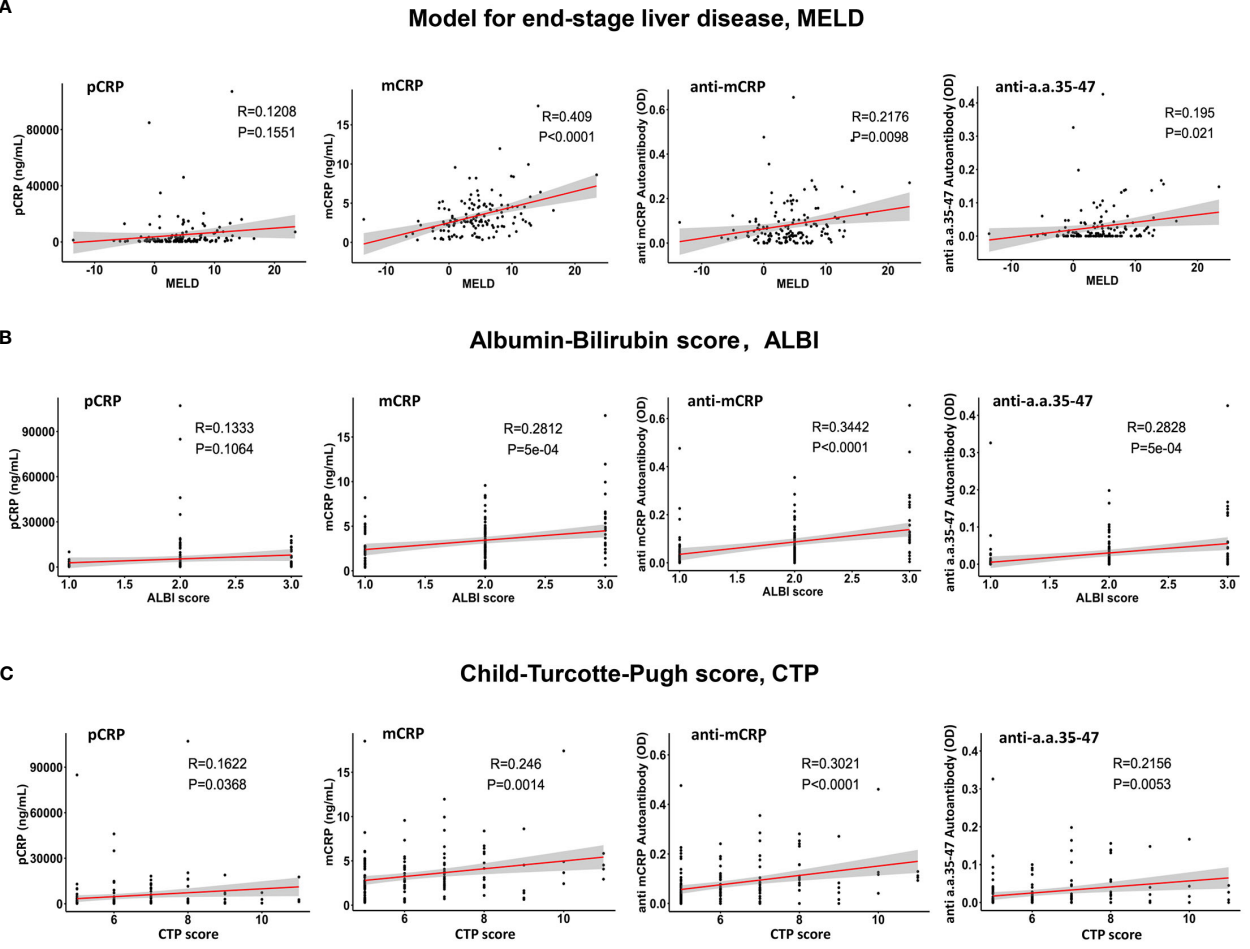
Analysis of the correlations of pCRP, mCRP, anti-mCRP autoantibodies, and anti-a.a.35-47 autoantibodies with the Model for End-Stage Liver Disease (MELD) **(A)**, albumin-bilirubin (ALBI) **(B)**, and Child–Turcotte–Pugh (CTP) **(C)** prognostic scores. The mCRP, anti-mCRP autoantibodies, and anti-a.a.35-47 autoantibodies were significantly positively correlated with all three scores, with mCRP exhibiting the highest correlation with MELD scores (r = 0.409; *p<* 0.0001), followed by anti-mCRP autoantibodies and anti-a.a.35-47 autoantibodies. CRP had a lower correlation with the CTP score and no correlation with the MELD and ALBI scores.

The association between pCRP, mCRP, anti-mCRP autoantibodies, and anti-a.a.35-47 autoantibodies with MELD, CTP, and ALBI scores was evaluated by Spearman correlation analysis. The results showed that mCRP had the highest correlation with MELD score (r = 0.409, *p<* 0.0001), followed by anti-mCRP autoantibodies and anti- a.a.35-47 autoantibodies; the correlation between pCRP and MELD score was not significant (r = 0.1208, *p* = 0.1551) (**Figure 2A**). Anti-mCRP autoantibodies (r = 0.3021, *p*< 0.0001), mCRP (r = 0.246, *p* = 0.0014), and anti-a.a.35-47 autoantibodies (r = 0.2156, *p* = 0.0053) were weakly correlated with CTP scores, whereas CRP was very weakly correlated with CTP score (**Figure 2B**). Anti-mCRP autoantibodies (r = 0.3442, *p<* 0.0001), anti-a.a.35-47 autoantibodies (r = 0.2828, *p* = 5 × 10^-4^), and mCRP (r = 0.2812, *p* = 5 × 10^-4^) were weakly correlated with ALBI scores, whereas CRP was not correlated with ALBI score (r = 0.1333, *p* = 0.1064) (**Figure 2C**).Overall, mCRP, anti-mCRP autoantibodies, and anti-a.a.35-47 autoantibodies showed significant positive correlations with all three scores, while pCRP showed a low correlation with CTP and no correlation with MELD or ALBI scores.

### 3.5 Association between pCRP, mCRP, anti-mCRP autoantibodies, and anti-a.a.35-47 autoantibodies and serum Alb and pre-Alb levels

**Figure 3:**
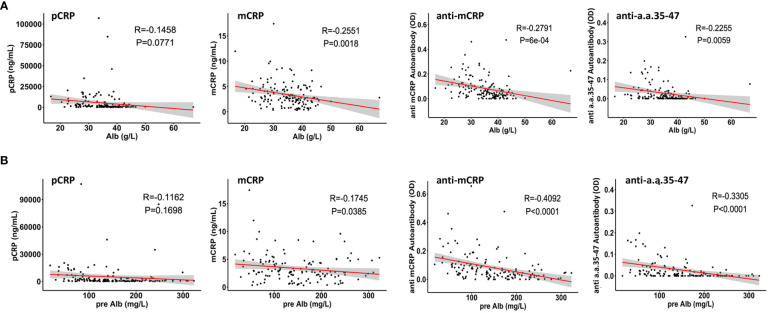
Correlations of pCRP, mCRP, anti-mCRP autoantibody, and anti-a.a.35-47 autoantibody levels with serum albumin (Alb) **(A)** and pre-albumin (pre-Alb) **(B)** levels. CRP was not correlated with Alb and pre-Alb. The levels of mCRP, anti-mCRP autoantibody, and anti-a.a.35-47 autoantibody were significantly negatively correlated with Alb and pre-Alb.

The correlations between pCRP, mCRP, anti-mCRP autoantibodies, and anti-a.a.35-47 autoantibodies with serum Alb and pre-Alb levels were analyzed. The results showed that except for pCRP, which did not correlate with Alb and pre-Alb, the other three indicators showed significant negative correlations with Alb and pre-Alb, with anti-mCRP autoantibodies having the highest negative correlation with pre-Alb (r = - 0.4092, *p<* 0.0001) (**Figure 3**). Previous studies showed that maintaining serum Alb concentration above 40 g/L may result in better patient prognosis (36), whereas elevated mCRP, anti-mCRP autoantibodies, and anti-a.a.35-47 autoantibodies implied an increased likelihood of poor prognosis. A persistent decline in Alb was suggestive of progressively aggravated necrosis of hepatocytes and poor prognosis, whereas increased Alb levels after treatment were suggestive of hepatocyte regeneration and therapeutic efficacy (36).

## 4 Discussion

HBV infection is one of the principal causes of end-stage liver diseases such as decompensated cirrhosis, severe hepatitis, advanced hepatocellular carcinoma, and liver failure (33). China has one of the greatest burdens of disease of cirrhosis worldwide. According to World Health Organization statistics, the proportion of patients with cirrhosis caused by HBV infection is about 30% worldwide, whereas the proportion of patients with cirrhosis caused by HBV infection in China is as high as 60% (37), and 15% of all cirrhosis-related deaths worldwide occurred in China (36, 38). Due to widespread hepatitis B vaccination, the number of new acute HBV infections in China has decreased substantially, but the number of patients with end-stage liver disease caused by HBV has increased annually along with the aging of the already-infected population. Therefore, it is essential to discover novel, non-invasive serum indicators that can be used to assist clinical diagnosis and treatment of patients with decompensated hepatitis B cirrhosis.

C-reactive protein is an inflammatory marker produced primarily by the liver as pentameric (pCRP) isoforms (39). mCRP is produced from the irreversible dissociation of pCRP in the local inflammatory microenvironment, which exposes a new epitope – the cholesterol binding sequence in amino acids 35-47 (CBS; a.a.35-47). pCRP is a weak predictor of infection and prognosis in patients with decompensated cirrhosis, but persistently elevated levels of pCRP may be indicative of patients with a higher short-term risk of death (40). In addition, there is growing evidence that pCRP depolymerizes in local tissues at the lesion to produce monomeric mCRP (16, 41), which is significantly more efficient in stimulating the inflammatory response, modulating complement system activation, and regulating LDL metabolism (42, 43). Thus, mCRP likely represents a “functional” isoform with significantly enhanced activity. However, whether monomeric forms of CRP and mCRP autoantibodies are associated with the severity and prognosis of liver disease remains unknown from existing studies. Therefore, in the present study, pCRP, mCRP, anti-mCRP autoantibodies, and anti-a.a.35-47 autoantibodies were studied simultaneously, and their serum levels and correlations with disease severity and theoretical prognosis were comprehensively evaluated and compared in patients with decompensated hepatitis B cirrhosis.

Preliminary results showed that serum pCRP, mCRP, and anti-mCRP autoantibody levels were significantly higher in patients in the decompensated hepatitis B cirrhosis group than in the healthy group; there was no significant difference in anti-a.a.35-47 autoantibodies. Among biochemical indicators at admission, ALT and AST were significantly higher in the high anti-mCRP autoantibody group than in the low anti-mCRP autoantibody group (*p* = 0.0057, *p* = 0.0174), AST was significantly higher in the high mCRP group than in the low mCRP group (*p* = 0.0183), and AST tended to be higher in the high CRP group than in the low CRP group (*p* = 0.0689) whereas there was no significant difference in ALT. Overall, liver function was reduced to different degrees in patients in the high-indicator groups. Among coagulation indicators at admission, the high anti-mCRP autoantibody group had higher PTR and INR and lower PTA and FIB than the low anti-mCRP autoantibody group, indicating that patients with high anti-mCRP autoantibody levels had worse coagulation function. There was no correlation between serum pCRP levels and any of the coagulation indicators. The correlation between pCRP, mCRP, anti-mCRP autoantibody, and anti-a.a.35-47 autoantibody levels and CTP, MELD, and ALBI scores were analyzed, and the results showed that mCRP, anti-mCRP autoantibodies, and anti-a.a.35-47 autoantibodies were significantly positively correlated with the three scores, with mCRP having the highest correlation with MELD score, followed by anti-mCRP autoantibodies and anti-a.a.35-47 autoantibodies, whereas pCRP had a low correlation with CTP score and no correlation with MELD and ALBI scores. In summary, the clinical correlation analysis and evaluation of the theoretical prognostic significance of pCRP, mCRP, anti-mCRP autoantibodies, and anti-a.a.35-47 autoantibodies indicated that mCRP and anti-mCRP autoantibodies may be more potentially advantageous as clinical indicators than pCRP with respect to disease status determination and prognostic value.

The present study was a preliminary investigation, and some limitations need to be acknowledged. First, the patient samples were from a single center and most of the study population was located in Northwest China, therefore, the influence of regional differences on the results cannot be completely excluded. Second, the lack of a compensated cirrhosis comparator cohort limits the implications that mCRP and anti-mCRP autoantibodies could be used to stratify disease severity or prognosis between the compensated and decompensated state. Third, the incidence of follow-up endpoint events in patients with decompensated hepatitis B cirrhosis was less than 10%, which did not allow for direct prognostic analysis. Instead, the prognostic scores of the theoretical predictions of the MELD, ABLI, and CTP systems were used, and actual prognostic analyses and validation of the theoretical prognostic models are currently unavailable. In future studies, we propose to further expand the sample size through a multicenter study and incorporate a longer follow-up period to increase the proportion of patients reaching the study endpoint for actual prognostic validation and improvement.

## Data availability statement

The original contributions presented in the study are included in the article/supplementary material. Further inquiries can be directed to the corresponding authors.

## Ethics statement

The studies involving humans were approved by the Ethics Committee of Xi’an Jiaotong University (2018059), and the study was conducted in accordance with the Declaration of Helsinki. The studies were conducted in accordance with the local legislation and institutional requirements. The participants provided their written informed consent to participate in this study.

## Author contributions

NG: Data curation, Investigation, Writing – original draft. PY: Investigation, Writing – original draft. ZT: Investigation, Writing – review & editing. JL: Investigation, Writing – review & editing. ZeY: Investigation, Writing – review & editing. MA: Writing – review & editing, Formal analysis. ZhY: Writing – review & editing, Formal analysis. DL: Writing – review & editing, Formal analysis. YW: Writing – review & editing, Conceptualization, Project administration, Supervision. HL: Project administration, Writing – review & editing, Funding acquisition, Writing – original draft.
